# No Apparent Costs for Facultative Antibiotic Production by the Soil Bacterium *Pseudomonas fluorescens* Pf0-1

**DOI:** 10.1371/journal.pone.0027266

**Published:** 2011-11-16

**Authors:** Paolina Garbeva, Olaf Tyc, Mitja N. P. Remus-Emsermann, Annemieke van der Wal, Michiel Vos, Mark Silby, Wietse de Boer

**Affiliations:** 1 Department of Microbial Ecology, Netherlands Institute of Ecology (NIOO-KNAW), Wageningen, The Netherlands; 2 Department of Biology, University of Massachusetts Dartmouth, North Dartmouth, Massachusetts, United States of America; 3 Department of Molecular Biology and Microbiology, Tufts University School of Medicine, Boston, Massachusetts, United States of America; Cairo University, Egypt

## Abstract

**Background:**

Many soil-inhabiting bacteria are known to produce secondary metabolites that can suppress microorganisms competing for the same resources. The production of antimicrobial compounds is expected to incur fitness costs for the producing bacteria. Such costs form the basis for models on the co-existence of antibiotic-producing and non-antibiotic producing strains. However, so far studies quantifying the costs of antibiotic production by bacteria are scarce. The current study reports on possible costs, for antibiotic production by *Pseudomonas fluorescens* Pf0-1, a soil bacterium that is induced to produce a broad-spectrum antibiotic when it is confronted with non-related bacterial competitors or supernatants of their cultures.

**Methodology and Principal Findings:**

We measured the possible cost of antibiotic production for *Pseudomonas fluorescens* Pf0-1 by monitoring changes in growth rate with and without induction of antibiotic production by supernatant of a bacterial competitor, namely *Pedobacter* sp.. Experiments were performed in liquid as well as on semi-solid media under nutrient-limited conditions that are expected to most clearly reveal fitness costs. Our results did not reveal any significant costs for production of antibiotics by *Pseudomonas fluorescens* Pf0-1. Comparison of growth rates of the antibiotic-producing wild-type cells with those of non-antibiotic producing mutants did not reveal costs of antibiotic production either.

**Significance:**

Based on our findings we propose that the facultative production of antibiotics might not be selected to mitigate metabolic costs, but instead might be advantageous because it limits the risk of competitors evolving resistance, or even the risk of competitors feeding on the compounds produced.

## Introduction

Interference competition is an important strategy of bacterial strains to establish and maintain themselves within microbial communities [1]. A well-known mechanism of bacterial interference competition is the production of antibiotics [2]. These secondary metabolites can be targeted against more or less closely related strains and species (e.g. bacteriocins) or against a wide range of competitors (e.g many polyketides) [3], [4], [5]. Theoretical models have demonstrated that, instead of decreasing diversity by leaving only the most aggressive strains, microbial warfare could actually promote diversity, with dynamic coexistence of many strains differing in their antibiotic production and sensitivity profiles [1], [6], [7], [8]. These results are obtained when it is assumed that both resistance to- and production of antibiotics come at a fitness cost, resulting in a reduced growth rate. The ecological trade-offs involved in investment in killing, resisting or outgrowing competing strains is thus predicted to maintain diversity.

Because of its profound relevance to human health, the fitness cost of bacterial resistance to antibiotics has received far more attention than the fitness cost of bacterial antibiotic production. Whereas it has emerged that the majority of bacterial antibiotic resistance mechanisms comes at a fitness cost [9], as predicted by theory [10], few studies have examined the cost of antibiotic production. Indications for biological costs of antibiotic production or antibiotic resistance are generally obtained by comparison of relative fitness of wild-type strains with that of antibiotic-negative mutant strains (e.g. [11], [12]). However, mutations causing loss of antibiotic production may cause additional changes in the bacterial phenotype [13], [14].

Previously, we have reported on competitor-induced triggering of broad-spectrum antibiotic production in fluorescent pseudomonads [15], [16]. The soil isolate *Pseudomonas fluorescens* Pf0-1 exhibits antibiotic activity only when it is confronted with specific phylogenetically unrelated competitors (e.g. *Pedobacter* sp.) or their supernatant indicating that it can distinguish interspecific competition from intraspecific competition [16]. Although the structure of the antimicrobial compound has not yet been elucidated, upregulated genes during confrontation with competitors point at the synthesis of a polyketide-like compound.[16]. In addition, we have shown that it has broad-spectrum activity, acting against both Gram-positive and Gram-negative bacteria as well as against fungi [16].

Competitor-dependent induction of antibiotic production allows for another possibility to examine costs of antibiotic production namely by comparing growth rates of wild-type bacteria with and without induction of antibiotic production. In the current study we used both approaches to quantify the possible fitness cost of antibiotic production in *P. fluorescens* Pf0-1: 1) comparison of the growth rate of the wild-type with and without induction of antibiotic production and 2) comparison of the growth rate of wild-type and non-antibiotic producing mutants under conditions that induce antibiotic production. All experiments were performed using nutrient-poor media, as soil-dwelling bacteria typically experience a scarcity of easily degradable carbon resources [17], [18]. Moreover, growth limiting conditions represent a situation in which the costs of antibiotic production should be most pronounced as there is no surplus of energy resources [19]. Costs were measured in liquid culture as well as on semi-solid medium in an incubation chamber, allowing quantification of micro-colony growth.

## Materials and Methods

### Bacterial and fungal cultures used

The bacterial strains used in this study are *Pseudomonas fluorescens* Pf0-1 (Gamma-proteobacteria) which was isolated from an agricultural loam soil in Sherborn, Massachusetts, USA [20] and *Pedobacter* sp. V48 (Sphingobacteria), which was isolated from a coastal dune site in The Netherlands [21]. The strains were pre-cultured from frozen glycerol stocks on 1/10 strength Tryptic Soy Broth agar (TSBA) [15]. In addition to the parental strain, deletion mutant ΔPfl01_3463-66 (with deletion of Pfl01_3463-3464: two branched-chain alpha-keto acid dehydrogenase E1 components; Pfl01_3465: branched-chain alpha-keto acid dehydrogenase subunit E2 and Pfl01_3466: dihydrolipoamide dehydrogenase) which is unable to produce the broad-spectrum antibiotic was used as well [16]. The fungal isolate *Rhizoctonia solani* anastomosis group 2.2IIIB, a plant-pathogenic basidiomycete, was used as bioindicator for production of broad-spectrum antimicrobial compounds [16].

### Preparation of cell-free *Pedobacter* supernatant

Cell-free supernatant was prepared by centrifugation (16,000 x g for 5 min) followed by filtration (Spin-X 0.22 µm filters; Corning Costar, Cat# 8160) of over-night cultures of *Pedobacter* strain V48 grown in 1/10^th^ strength Tryptic Soy Broth at 20^o^C. An aliquot of the cell-free supernatant was boiled for 10 minutes and was used as a control. Boiling the cell-free supernatant for 10 minutes was sufficient to destroy the signalling compound(s) that trigger antibiotic production in *P. fluorescens* Pf0-1.

### Determination of growth of Pf0-1 in nutrient-poor liquid media

The effect of cell-free supernatant from *Pedobacter* sp V48 on the growth rate of Pf0-1 was determined in a nutrient-poor liquid medium (0.5 gL^−1^ KH_2_PO_4;_ 0.1 gL^−1^ BD Bacto^TM^ Yeast extract (Cat# 210934) and 0.1 gL^−1^(NH_4_)_2_SO_4_; adjusted to pH 6.5). This medium was supplemented with 10% (v/v) boiled (control) or unboiled *Pedobacter* supernatant, respectively. *P. fluorescens* Pf0-1 was inoculated to an optical density (OD) (600 nm) of 0.02 which corresponds to 2.6×10^6^ cells/ml^−1^. The 50 ml cultures were incubated for 24 hours at 20°C shaken at 200 rpm and 1 ml samples were taken every hour for OD measurements and viable counts. Additionally, the growth rate of *P. fluorescens* Pf0-1 wild-type and mutants ΔPfl01_3463 (both supplemented with 10% (v/v) boiled (control) or unboiled *Pedobacter* supernatant) were measured in 96-well plates (Greiner bio-one, Cat# 655180) using Synergy Microplate Reader. The OD measurements were performed every 30 minutes for total period of 8.5 hours.

### Agar-incubation chambers for observation of bacterial growth on semi-solid medium

**Figure 1:**
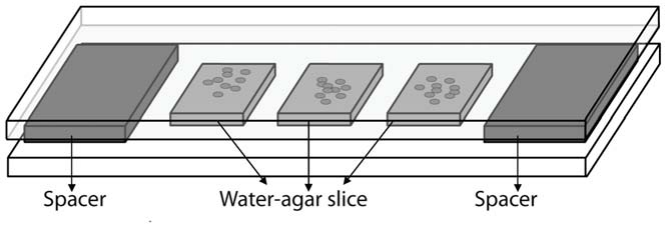
Incubation chamber for determination of bacterial growth made of a glass slide with cardboard spacers on both sides. Between the spacers 3 pre-inoculated water-agar (WA) slices of 1 cm^2^ are placed. A glass coverslip is slightly pressed on top of the agar slices. The sides of the incubation chamber are sealed with parafilm.

1 ml of *P. fluorescens* Pf0-1 overnight culture was centrifuged for 3 min at 16,000 g. The cells were resuspended in 10 mM phosphate buffer (pH 6.5) containing 10% non-boiled or boiled cell-free supernatant of *Pedobacter* sp V48 to an optical density of 0.002 at 600 nm; so, the initial number of cells per ml was 10 times lower than in the liquid growth experiments. The lower number was used as it allows distinguishing individual bacteria on the agar slices (see below). An aliquot of 280 µl of *P. fluorescens* Pf0-1 bacterial suspension was equally spread on a thin layer (2 mm) of water agar containing 5 g L^−1^ NaCl; 1 g L^−1^ KH_2_PO_4_ and 0.1g L^−1^ (NH_4_)_2_SO_4_ adjusted to pH 6.5 as previously described [15]. Since bacterial growth was observed on this water-agar medium no extra substrate (yeast extract) was added [16]. Apparently, the substrates that were added with the 10% *Pedobacter* supernatant and the substrates that became available after autoclaving of agar were sufficient for growth. After inoculation the plates were dried for about 5 minutes in a flow cabinet and 3 slices of 1 cm^2^ were cut and carefully transferred to incubation chambers. The incubation chamber described in Figure 1 is adapted from Reinhard and Van der Meer [22] and Robin Tecon (unpublished data).

### Microscopical counting and data analysis

Twenty randomized pictures at 400-fold magnification representing an area of 1 mm^2^ were taken of each slice with an Axio Imager M1 microscope (Carl Zeiss, Oberkochen, Germany) under phase-contrast illumination and an AxioCam MRm camera. Microscopic enumerations were performed every hour for a period of 9 hours at room temperature. During this period microcolonies remained two-dimensional, i.e. no stacking took place. Digital images were analyzed using the AXIO VISION v4.7 Software (Carl Zeiss Imaging Solutions GmbH, Germany) for enumeration and area determination of bacterial colonies.

### Extraction of antimicrobial compound and test for inhibition

*P. fluorescens* Pf0-1 overnight liquid cultures exposed to 10% boiled or unboiled supernatant of *Pedobacter* sp V48 (initial volume 35 ml) were acidified with trifluoroacetic acid (0.1% (v/v)), mixed with 2 volumes of ethylacetate and shaken vigorously for 5 min. as described by Raaijmakers et al. [23]. After overnight incubation at −20°C the unfrozen liquid (ethylacetate) fraction containing the active compounds was carefully transferred to a new flask and dried under constant air-flow. The dried extract was dissolved in 150 µl 50% (v/v) methanol and tested for inhibition of the bacterial isolate *Pedobacter* sp V48 and the soil borne plant-pathogenic fungus *Rhizoctonia solani* as described by Garbeva et al. [16].

### RNA extraction, cDNA synthesis and real-time PCR

RNA was extracted from *P. fluorescens* Pf0-1 grown in nutrient-poor liquid medium that had been exposed to 10% boiled or non-boiled supernatant of *Pedobacter* sp V48 as described above. Cells for RNA extraction were collected at 4 time points (3 h, 6 h, 8 h and 24 h) and diluted with sterile phosphate buffer to the same optical density (600 nm) to obtain equal amounts of cells. The RNA extraction and cDNA synthesis were performed as described previously by Garbeva et al [16].

For real-time PCR assessment of expression of genes involved in antibiotic production two different primer combinations were used: (1) primer combination 3463F835 (GAT TTT TAC GCG GTC TAC GC) and 3463R1036 (TGA TCA GGT TGC TGT TTC AGG) amplifying 206 bp from gene Pf01_3463 encoding the two branched-chain alpha-keto acid dehydrogenase E1 component and (2) primer combination 3465T1F (CAG GGC CCG ATG GTT GC) and 3465T1R (TTG CTT TTT GTG CCG CGC TCG) amplifying 348 bp from gene Pf01_3465 encoding branched-chain alpha-keto acid dehydrogenase subunit E2. As a control, a 210 bp fragment of the house keeping 16S rDNA gene was amplified using primer combination 16SPf01F (TTG GGA GCC TTG AGC TCT TA) and 16SPf01R (AAG GCA CCA ATC CAT CTC TG). Real-time PCR was performed using a Corbett Research Rotor-Gene 3000 thermal cycler (Westburg, Leusden, The Netherlands) with the following conditions: an initial cycle of 95°C for 15 min followed by 40 cycles of: 95°C for 15 sec; 56°C for 50sec and 72°C for 50 sec. Standard curves were established for each primer combination.

### Statistical analysis

All experiments were performed in triplicate. Differences in optical densities of liquid cultures between treatments were tested for significance for each time interval by one-way analysis of variance. Bacterial viable count data that were used to calculate the maximum yield (maximum number of colony forming units per ml) were also log-transformed prior to one-way analysis of variance. For the analysis of microcolony development the data were log transformed and the slopes of the regression lines were compared in GraphPad Prism 5 (GraphPad Software, Inc., CA, USA) using a two tailed t-test. The statistical analyses of quantitative real-time PCR data were carried out with XLStat 2010 (Addinsoft, New York, USA) using a two-tailed t-test. Data were considered to be statistically different at p≤0.05.

## Results

### *P. fluorescens* Pf0-1 antibiotic production and growth rate

**Figure 2:**
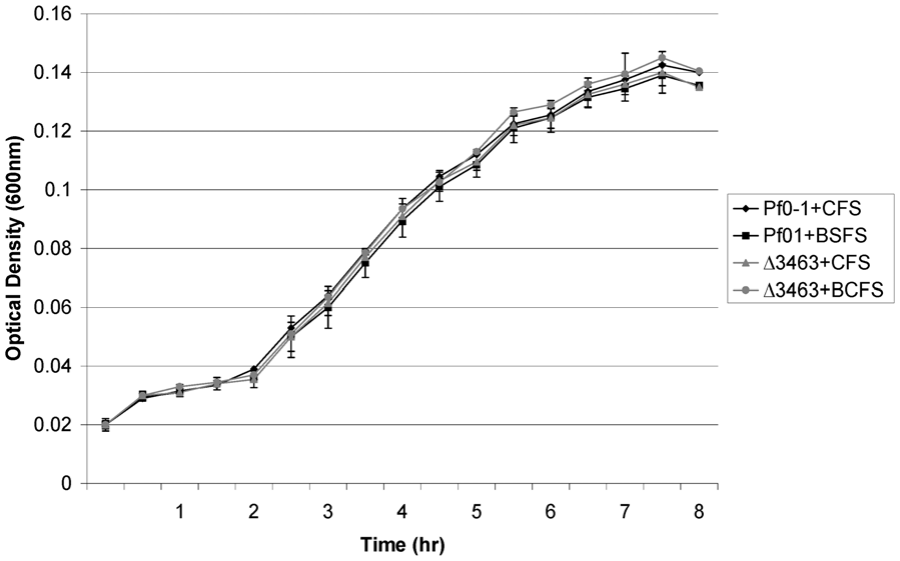
Bacterial growth (Optical Density at 600 nm) in nutrient-poor liquid medium in microplates. : Pf01wt+CFS - *P. fluorescens* Pf0-1 wild type with 10% cell-free supernatant from *Pedobacter* sp. V48; Pf01wt+BCFS - *P. fluorescens* Pf0-1 wild type with 10% boiled cell-free supernatant from *Pedobacter* sp. V48; Δ3463+CFS – *P. fluorescens* Pf0-1 mutant Δ3463 with 10% cell-free supernatant from *Pedobacter* sp. V48 and Δ3463+BCFS – *P. fluorescens* Pf0-1 mutant Δ3463 with 10% boiled cell-free supernatant from *Pedobacter* sp. V48. The measurements were performed every 30 min for total period of 8.5 h. Symbols represent means of 3 replicate measurements; error bars represent standard deviations.

The OD measurements indicated that Pf0-1 grew only a short period (1–2 hr) exponentially in nutrient-poor liquid media followed by linear increase (Figure 2; Figure S1). This rapid decline in growth rates occurred for all treatments and stationary phase was reached after 8 hours. At none of the time intervals (performed on 96-well plates every 30 min) were there significant differences in OD between cultures exposed to boiled and unboiled *Pedobacter* supernatant (Figure 2). The growth rate of Pf0-1 mutant Δ3463, which is deficient in the production of the broad-spectrum antimicrobial compound triggered by *Pedobacter* supernatant, was also compared with that of the wild-type strain in the presence of *Pedobacter* supernatant. Again, no differences in OD were observed (Figure 2).

Similar observations were made for the 50 ml cultures where OD measurements were performed every hour (Figure S1). For the latter cultures, the maximum yield (highest number of viable cells per ml of growth medium) was calculated and again no differences were found between the treatments (Table 1.).

**Table 1:** Maximum yield of *P. fluorescens* Pf0-1 wild-type and mutant Δ3463 strains in nutrient-poor liquid medium.

Treatment	Maximum yield * (CFU per ml)
Pf0-1 wild-type+10% cell-free *Pedobacter* supernatant	7.99±0.013
Pf0-1 wild-type+10% boiled cell-free *Pedobacter* supernatant	7.96±0.022
Δ3463+10% cell-free *Pedobacter* supernatant	8.00±0.021
Δ3463+10% boiled cell-free *Pedobacter* supernatant	7.99±0.016

*The maximum yield calculated at time point  = 7.5 h is the mean of tree replicates per treatment. The variation between the replicates is indicated as ± s.d.

One-way ANOVA did not reveal difference between the treatments p>0.05.

The liquid cultures used for growth rate determinations were extracted at the end of the incubation to confirm that the wild-type Pf0-1 was induced by *Pedobacter* supernatant to produce antibiotics. The presence of antibiotic activity in the extracts was tested by determining the effect of the extracts on growth of *Pedobacter* sp V48 and the soil-borne pathogenic fungus *Rhizoctonia solani*. Inhibition of *Rhizoctonia* and *Pedobacter* was observed only with the extracts from wild type Pf0-1 cultures exposed to 10% cell-free *Pedobacter* supernatant (Figure S2A, B). There was no such inhibition by the extracts obtained from Pf0-1 cultures exposed to 10% boiled cell-free *Pedobacter* supernatant or from Pf0-1 mutant Δ3463 cultures exposed to 10% cell-free *Pedobacter* supernatant.

### Expression of genes involved in antimicrobial compound production

At four time points (t = 3 h, 6 h, 8 h and 24 h) during growth in liquid medium, *P. fluorescens* Pf0-1 cells were collected for RNA isolation, cDNA synthesis and quantitative RT-PCR. Primers targeting genes Pfl01_3463 and Pfl01_3465 were used for quantitative RT-PCR. Genes Pfl01_3463 and Pfl01_3465 encode branch-chain alpha-keto acid dehydrogenase E1 components and branched-chain alpha-keto acid dehydrogenase subunit E2 that were previously demonstrated to be essential for the production of broad-spectrum antimicrobial activity by *P. fluorescens* Pf0-1 [16]. Quantitative RT-PCR revealed that triggering of Pfl01_3463 and Pfl01_3465 genes expression by *Pedobacter* supernatant was already apparent after three hours, as there was a significant (3.16 and 2.57 fold respectively) increase in comparison with the expression of these genes in cultures of Pf0-1 exposed to 10% boiled *Pedobacter* supernatant (Table 2). The expression of genes Pfl01_3463 and Pfl01_3465 was always at least two-fold higher in the Pf0-1 cultures exposed to 10% cell-free *Pedobacter* supernatant than in the controls (Pf0-1 cultures exposed to 10% boiled cell-free *Pedobacter* supernatant).

**Table 2:** Quantitative real-time PCR comparison of gene expression in *P. fluorescence* Pf0-1 with triggered antibiotic production (treatment) and non-triggered antibiotic production (control).

Time points hours	Fold change treatment/ to control *gene Pf01_3463*	Fold change treatment/ to control *gene Pf01_3465*
3	3.16±0.03	2.57±0.11
6	2.39±0.06	2.68±0.03
8	2.11±0.11	3.21±0.14
24	2.50±0.10	2.6±0.10

Treatment- *P. fluorescence* Pf0-1 with 10% cell-free supernatant of *Pedobacter* sp. V48 and control- - *P. fluorescence* Pf0-1 with 10% boiled cell-free supernatant of *Pedobacter* sp. V48.

Differential expression is given as fold-changes treatment relative to the control.

The numbers are the means of tree replicates per treatment. The variation between the replicates is given as ± s.d.

All fold changes of gene expression in the treatment were significantly different (p<0.05) from the control.

### Effect of antibiotic production on *P. fluorescens* Pf0-1 growth rate on agar and microcolony morphology

**Figure 3:**
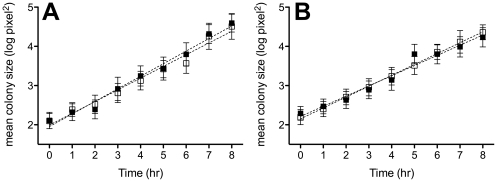
Bacterial growth in micro-colonies measured microscopically using Water-Agar incubation chambers. (A) white squares -wild type *P. fluorescens* Pf0-1 exposed to 10% cell-free supernatant from *Pedobacter* sp. V48 and black squares- wild type *P. fluorescens* Pf0-1 exposed to 10% boiled cell-free supernatant from *Pedobacter* sp. V48; (B) white squares -wild type *P. fluorescens* Pf0-1 exposed to 10% cell-free supernatant from *Pedobacter* sp. V48 and black squares- mutant Δ3463 exposed to 10% cell-free supernatant from *Pedobacter* sp. V48. Squares represent the means of micro-colony sizes and the error bars represent the standard deviations. Statistical analysis revealed no significant differences (p>0.05) between the growth rates (slopes) of the treatments.

**Figure 4:**
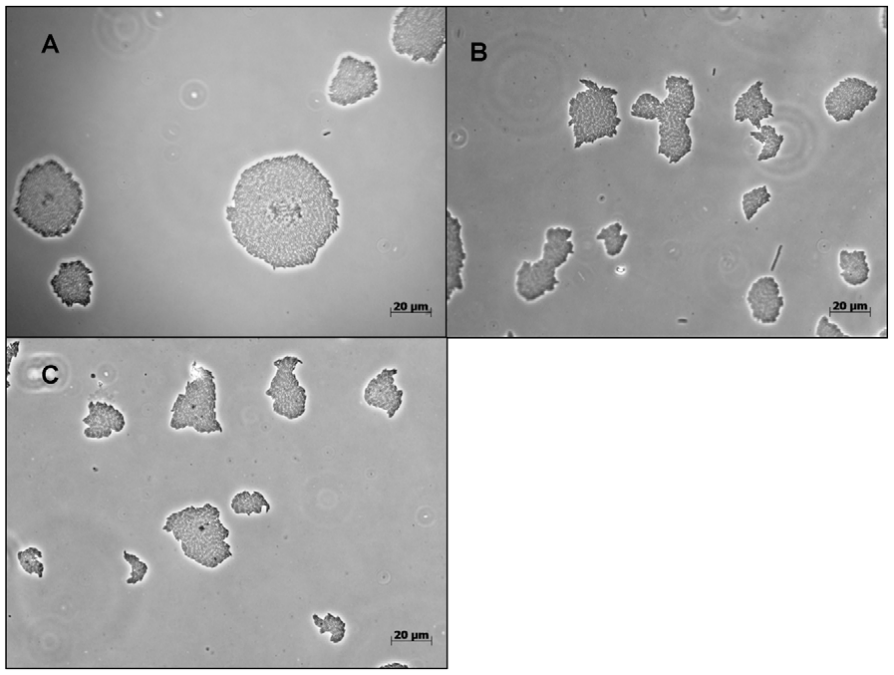
Morphology of bacterial micro-colonies after 7 h of incubation on Water-Agar of (A) wild type *P. fluorescens* Pf0-1 exposed to 10% cell-free supernatant from *Pedobacter* sp. V48 (B) wild type *P. fluorescens* Pf0-1 exposed to 10% boiled cell-free supernatant from *Pedobacter* sp. V48 and (C) - Mutant Δ3463 exposed to 10% cell-free supernatant from *Pedobacter* sp. V48.

Using water-agar incubation chambers we determined growth rate and size of Pf0-1 colonies with and without induction of antibiotic production by *Pedobacter* supernatant. Growth (increase of surface areas micro-colonies) was exponential during the period of examination (Figure 3). There was no significant difference in growth rates between the two treatments (Figure 3A). No significant differences in growth rates were observed between the colonies of wild type and mutant Δ3463 strains either (Figure 3B). In fact, the average colony size of Pf0-1 supplied with 10% cell-free supernatant from *Pedobacter* sp V48 was slightly (but not significantly) bigger than that of Pf0-1 supplied with boiled cell-free supernatant. However, there was a clear difference in colony morphology. After 7 hours of incubation, wild-type Pf0-1 exposed to 10% *Pedobacter* supernatant started to form spherical colonies, a phenomenon not observed for Pf0-1 exposed to boiled *Pedobacter* supernatant or for Pf0-1 mutant Δ3463 exposed to unboiled *Pedobacter* supernatant (Figure 4).

## Discussion

Many, if not most, bacteria produce a range of secondary metabolites that can target competing microorganisms [2], [5]. Natural bacterial populations have been found to consist of a wide variety of genotypes that differ in their ability to both suppress and withstand conspecifics [4], [24], [25], [26]. A trade-off between an advantage in growth (resource or scramble competition) and an advantage in ‘killing capacity’ or ‘resistance capacity’ (interference competition) lies at the basis of theoretical models attempting to explain the coexistence of strains differing in antibiotic production and sensitivity [1], [6], [7], [8], [27], [28]. The reason that not all strains evolve to produce antibiotics thus is explained by the fact that the production of these compounds (and their corresponding immunity factors) incurs a metabolic cost. In competition in a structured environment, antibiotic producing cells will displace sensitive (non-antibiotic producing) cells, whereas sensitive (non-antibiotic producing) cells have a growth rate advantage over resistant (non-antibiotic producing) cells that in turn displace antibiotic producing cells because they do not carry the cost of antibiotic production [29].

Previously, it was hypothesized that the facultative- rather than the constitutive production of antibiotics represents a cost-effective strategy, as the antibiotic compound is only produced in situations where it is needed [16]. Our finding that the cost of antibiotic production in the *P. fluorescens* Pf0-1 system is not significant is not in line with the cost-based assumption on basis of which theoretical models aim to explain how microbial warfare can promote microbial diversity. If these costs are truly insignificant then why do not all strains produce antimicrobial compounds constitutively? Two alternative ecological trade-offs could be envisaged to be at work. First, facultative antibiotic production could prevent competitors evolving resistance to the antibiotic by reducing exposure [16]. It is evident from clinical studies that increased exposure to antibiotics (through patient consumption) can result in higher resistance levels in a pathogen population (e.g. [30]). Second, it has recently emerged that many bacteria can actually subsist on antibiotic compounds [31], [32]. Although antibiotic production could inhibit the growth of some strains competing for resources, it could promote the growth of others. It is presently unknown how important both mechanisms are in bacterial populations in soil but they certainly seem worthy of future attention.

Whilst we did not observe significant costs of antibiotic production here, biological costs associated with antibiotic resistance have been reported to vary from significant (e.g. [9], [33], [34]), to no-cost (e.g. [14], [35], [36], [37]) to even enhanced fitness (e.g. [37]). This variation in costs might be explained by the fact that the genetic systems underlying antibiotic resistance are diverse and furthermore might not be readily comparable to those that underlie antibiotic production (including the production of a molecule conferring autoimmunity). Mutations conferring resistance arising in sensitive cells often modify the molecule targeted by the antibiotic in such a way that, although the cell is protected from the antibiotic, its function is severely compromised [9]. Such non-additive, pleiotropic fitness costs often are severe. With antibiotic production on the other hand, it could be hypothesized that the cells mainly bear the additive, metabolic cost of the production of the antibiotic and the immunity molecules (which must be small compared to the sum of all other molecules produced by the cell) and suffer less from pleiotropic costs. In addition, compensatory mutations that mitigate pleiotropic fitness costs have been identified in resistant bacteria [9]. It could be that compensatory mutations are more readily accessible for genetic systems mediating antibiotic production than they are for resistance mutations thus lowering the fitness cost in the former.

Although we did not observe costs for antibiotic production, clear changes in *P. fluorescens* Pf0-1 colony morphology during antibiotic production were apparent from the agar chamber experiments. Antibiotic producing *P. fluorescens* Pf0-1 cells formed spherical colonies whereas the non-producing mutant as well as wild-type strain in the control situation did form irregular shaped colonies. Recently it was reported that different *P. fluorescens* colony morphology variants have distinct metabolic profiles [38]. It seems plausible that the spherical colony-shape of Pf0-1 here is a response to the produced antibiotic and not to a signal of *Pedobacter,* as the mutant deficient in the production of the antibiotics did not produce spherical colonies in the presence of *Pedobacter* supernatant. Formation of such spherical colonies may coincide with antibiotic production to obtain the highest protection against antagonising organisms. Further studies are needed to understand the mechanism and the biological relevance of changes in colony morphology of *P. fluorescens* Pf0-1 during antibiotic production.

## Supporting Information

Figure S1 **Bacterial growth (Optical Density 600 nm) in 50 ml nutrient-poor liquid cultures.**: Pf01wt+CFS - wild type *P. fluorescens* Pf0-1 with 10% cell-free supernatant from *Pedobacter* sp. V48; Pf01wt+BCFS - wild type *P. fluorescens* Pf0-1 with 10% boiled cell-free supernatant from *Pedobacter* sp. V48; Δ3463+CFS – *P. fluorescens* Pf0-1 mutant Δ3463 with 10% cell-free supernatant from *Pedobacter* sp. V48. The measurements are presented as mean ± SD (n = 3 replicates).
(TIF)

Figure S2 (A) Antagonist assay against the fungus *Rhizoctonia solani* with extracts from liquid cultures (Figure S1) 1- Mutant Δ3463 with 10% cell-free supernatant from *Pedobacter* sp. V48; 2- wild type *P. fluorescens* Pf0-1 with 10% cell-free supernatant from *Pedobacter* sp. V48; 3- wild type *P. fluorescens* Pf0-1 with 10% boiled cell-free supernatant from *Pedobacter* sp. V48 and C- control 50% methanol. (B) Effect of different extracts from liquid cultures (Figure S1) on growth of *Pedobacter* sp. V48 (growing on 1/10 TSBA for 24 h) 1.Ext.wt- extract from wild type *P. fluorescens* Pf0-1 with 10% cell-free supernatant from *Pedobacter* sp. V48; 2. Ext.mut- extract from mutant Δ3463 with 10% cell-free supernatant from *Pedobacter* sp. V48 and 3.Con- extract from wild type *P. fluorescens* Pf0-1 with 10% boiled cell-free supernatant from *Pedobacter* sp. V48. 4. PdV48- *Pedobacter* sp. V48 without any extract. Data are presented as mean ± SD (n = 3 replicates). * - Indicates significant reduction of *Pedobacter* sp. V48 CFU as compared to other treatments (p<0.05) as analyzed by one-way ANOVA followed by Tukeỳs HSD test.
(TIF)
