# Supplementary figures and images for: No Apparent Costs for Facultative Antibiotic Production by the Soil Bacterium Pseudomonas fluorescens Pf0-1
Source: PLoS One. 2011 Nov 16;6(11):e27266. doi: 10.1371/journal.pone.0027266 (PMC3217935; doi:10.1371/journal.pone.0027266)

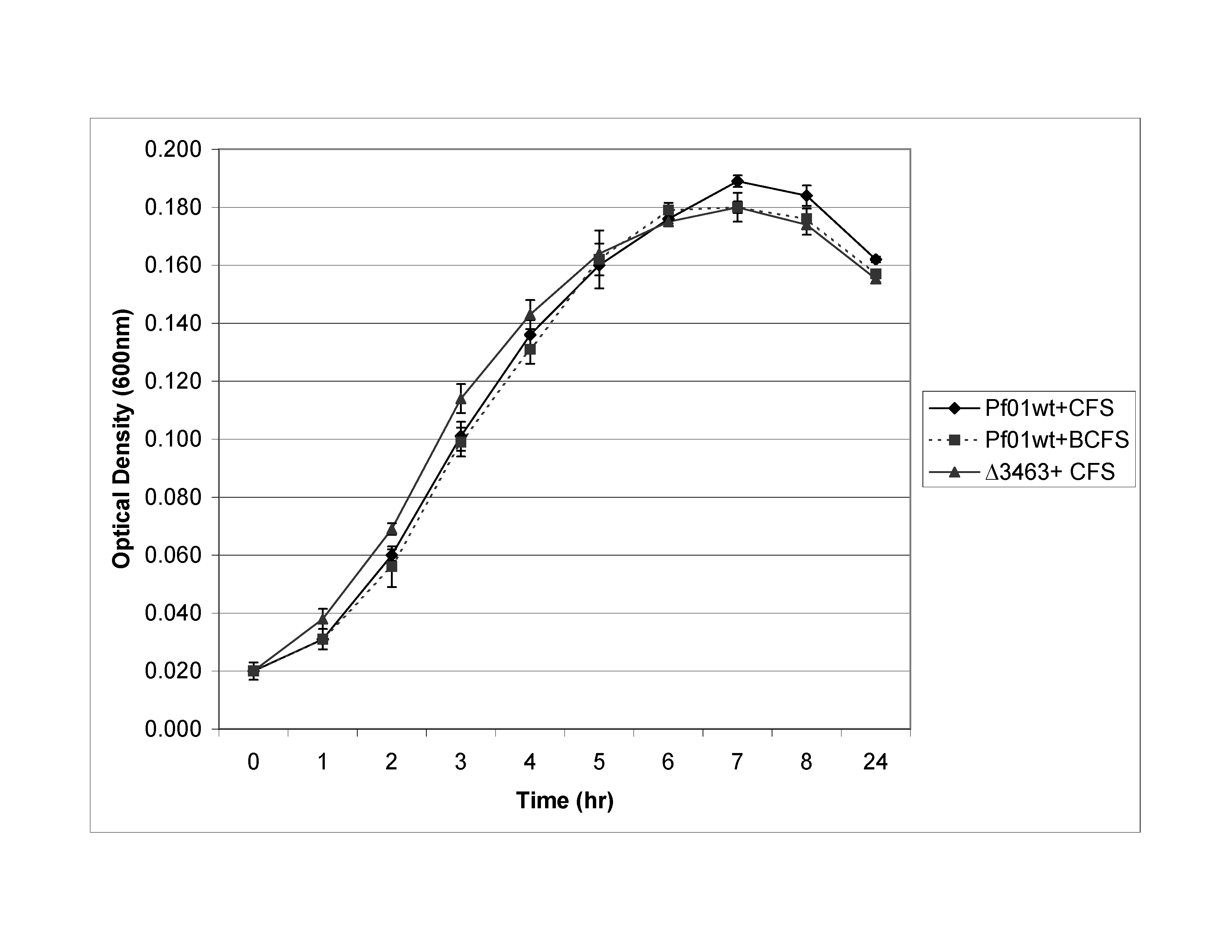

Supplement: Figure S1 — Bacterial growth (Optical Density 600 nm) in 50 ml nutrient-poor liquid cultures.: Pf01wt+CFS - wild type P. fluorescens Pf0-1 with 10% cell-free supernatant from Pedobacter sp. V48; Pf01wt+BCFS - wild type P. fluorescens Pf0-1 with 10% boiled cell-free supernatant from Pedobacter sp. V48; Δ3463+CFS – P. fluorescens Pf0-1 mutant Δ3463 with 10% cell-free supernatant from Pedobacter sp. V48. The measurements are presented as mean ± SD (n = 3 replicates). (TIF) [file pone.0027266.s001.tif]

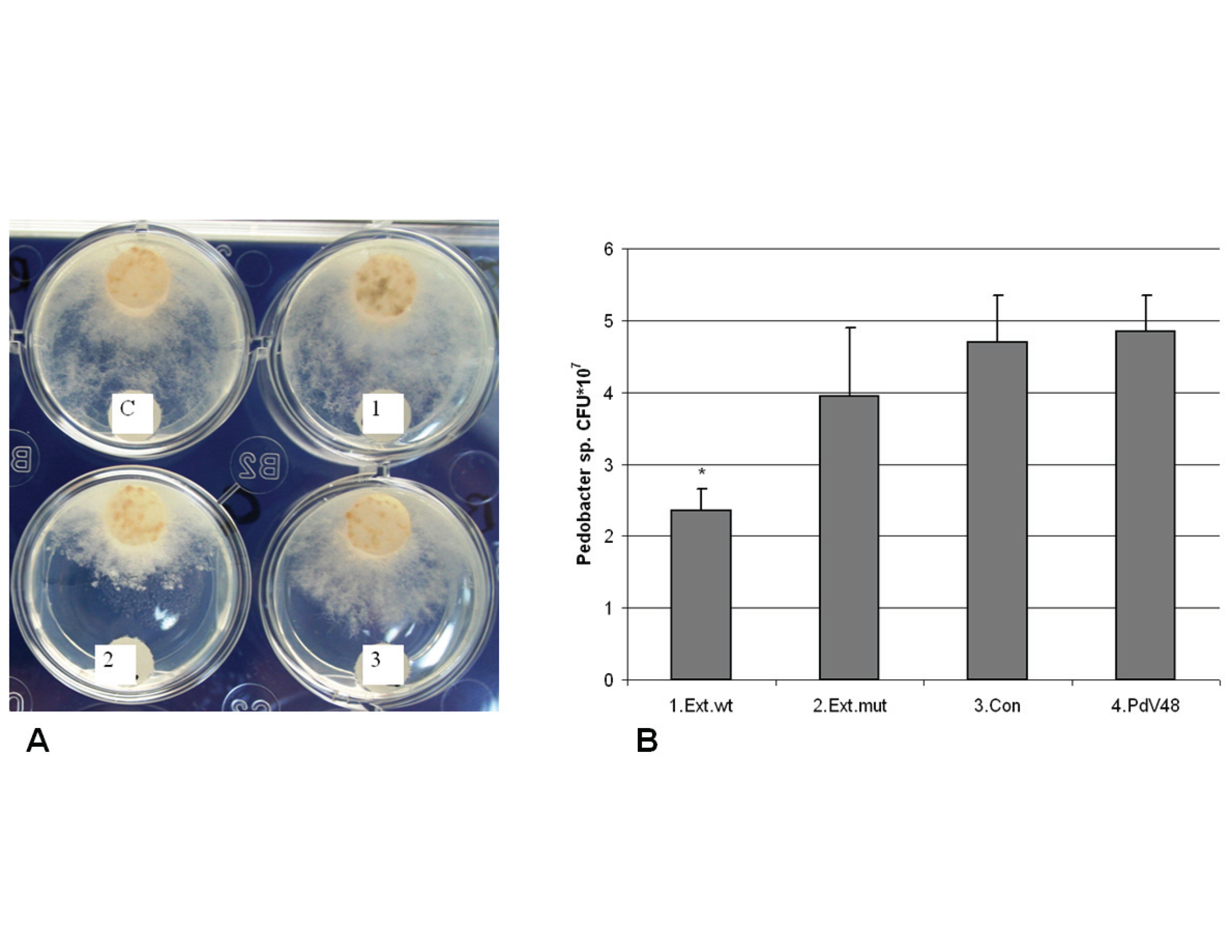

Supplement: Figure S2 — (A) Antagonist assay against the fungus Rhizoctonia solani with extracts from liquid cultures (Figure S1) 1- Mutant Δ3463 with 10% cell-free supernatant from Pedobacter sp. V48; 2- wild type P. fluorescens Pf0-1 with 10% cell-free supernatant from Pedobacter sp. V48; 3- wild type P. fluorescens Pf0-1 with 10% boiled cell-free supernatant from Pedobacter sp. V48 and C- control 50% methanol. (B) Effect of different extracts from liquid cultures (Figure S1) on growth of Pedobacter sp. V48 (growing on 1/10 TSBA for 24 h) 1.Ext.wt- extract from wild type P. fluorescens Pf0-1 with 10% cell-free supernatant from Pedobacter sp. V48; 2. Ext.mut- extract from mutant Δ3463 with 10% cell-free supernatant from Pedobacter sp. V48 and 3.Con- extract from wild type P. fluorescens Pf0-1 with 10% boiled cell-free supernatant from Pedobacter sp. V48. 4. PdV48- Pedobacter sp. V48 without any extract. Data are presented as mean ± SD (n = 3 replicates). * - Indicates significant reduction of Pedobacter sp. V48 CFU as compared to other treatments (p<0.05) as analyzed by one-way ANOVA followed by Tukeỳs HSD test. (TIF) [file pone.0027266.s002.tif]
